# Pediatric Celiac Disease in Central and East Asia: Current Knowledge and Prevalence

**DOI:** 10.3390/medicina55010011

**Published:** 2019-01-12

**Authors:** Dimitri Poddighe, Marzhan Rakhimzhanova, Yelena Marchenko, Carlo Catassi

**Affiliations:** 1 Department of Medicine, Nazarbayev University School of Medicine, 010000 Astana, Kazakhstan; 2 Department of Pediatrics, National Research Center of Mother and Child Health, University Medical Center, 010000 Astana, Kazakhstan; Marzhan.Rahimzhanova@umc.org.kz; 3 Center of Laboratory Medicine, Republican Diagnostic Center, University Medical Center, 010000 Astana, Kazakhstan; elena.martchenko@gmail.com; 4 Department of Pediatrics, Universita’ Politecnica delle Marche, 60121 Ancona, Italy; c.catassi@univpm.it

**Keywords:** celiac disease, children, HLA-DQ, prevalence, Asia

## Abstract

The current prevalence of pediatric Celiac Disease (CD) is estimated to be around 1% in the general population, worldwide. However, according to the geographic area, a great variability of CD prevalence has been described. Whereas a number of studies are available from Europe, North and South America, Australia, South-West Asia, and North Africa, the knowledge and awareness of CD in large parts of the remaining world areas is definitively poor. In several countries of Central and East Asia, the consumption of wheat is consistent and/or has significantly increased in recent decades, and CD is supposed to be underdiagnosed in children. In this mini-review, we aimed to summarize the current knowledge about the prevalence of pediatric CD in Central and East Asia, paying attention to the HLA-DQ immunogenetic background as well. Indeed, CD is likely not to be as uncommon as previously or currently thought in countries like Russia, Kazakhstan, and China, in addition to India, where pediatric CD has been clearly showed to be quite prevalent. Therefore, there is an urgent need for population-based studies on the prevalence of CD in those countries, especially in children, in order to increase the awareness of this disease and to improve the diagnostic strategy in these areas.

## 1. Introduction

Celiac disease (CD) is a multifactorial immune-mediated disorder, triggered by the ingestion of gluten and other gluten-related proteins in genetically predisposed subjects. HLA-DQ alleles, coding α and β chains of MHC-DQ2 and -DQ8 heterodimers, have been shown to be a necessary—but not sufficient—immunogenetic background for the development of CD. Moreover, gluten is the required environmental exposure to develop CD in genetically predisposed individuals, probably in addition to other variable constitutional and/or acquired factors [1,2,3].

CD is a systemic disease, as the clinical manifestations are not limited to the intestinal tract: indeed, it is estimated that a significant portion of CD cases are currently undiagnosed, and its great clinical heterogeneity with ‘atypical’ and variable extra-intestinal manifestations is undoubtedly a major contributing factor [4,5]. However, the growing awareness about CD among patients and physicians has drawn specific attention on this diagnosis and even prompted a great debate on widened screening strategies in Western countries. Because of this, the actual and estimated prevalence rates of CD have increased significantly in the last few decades [6,7]. Even in some countries where CD was considered to be nonexistent or negligible until recent years, this disease has been found to be quite frequent and, in some populations, recent immunogenetic analyses and epidemiological data suggested that CD prevalence and risk may be comparable to Europe and North America [8].

Despite a wide variation in prevalence according to the geographic area, pediatric CD is currently considered to affect almost 1% of the general population worldwide. Indeed, CD is frequent in Europe, North and South America, Australia, South-West Asia, and North Africa, where more than 30% of the population carry the DQ2 haplotype and high consumption of wheat is present. In contrast, CD is extremely rare in Far East Asia and in sub-Saharan Africa, where wheat and other gluten cereals are not staple foods [9].

As mentioned above, during the last few years several studies have also suggested CD to be common in South West Asia. Population screening studies have been carried out in Iran, Turkey and Israel, where the prevalence figured comparable to Europe [10,11]. However, in most other parts of Asia, data on CD prevalence are scarce or even totally absent. As the consumption of wheat is actually consistent in several of these areas [12], a high number of unrecognized CD cases may actually exist, and be undiagnosed because of poor awareness of this disease.

**Figure 1:**
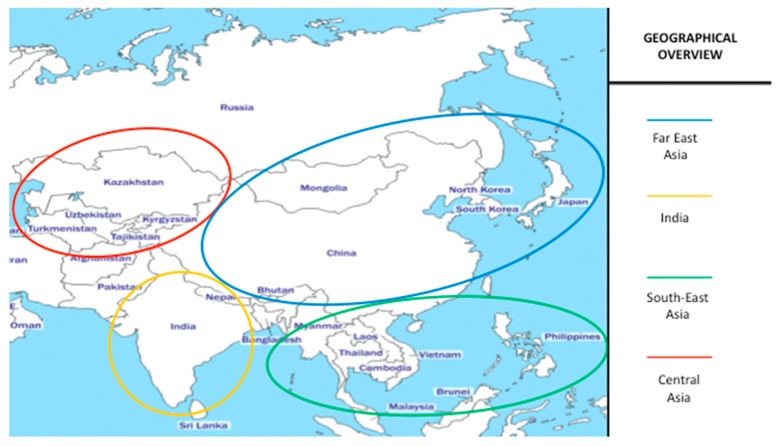
Overview of the geographical macro-areas of Central and East Asia, as discussed in each section of the review.

In this mini-review, we aim to summarize the current knowledge about pediatric CD in Central and East Asia, where very few studies and research are currently available through the Medline/Pubmed database, except for the Indian subcontinent. The specific Asian areas that have been discussed in each section of this review are graphically represented in Figure 1. This article organization by geographical macro-areas will help readers to understand and appreciate the wide differences in the knowledge of pediatric CD epidemiology and disease awareness among countries of non-Western Asia, where many more studies on this topic have been carried out so far.

## 2. Far East Asia

In Japan, the overall prevalence of the HLA-DQ2 and -DQ8 immunotypes has been reported to be quite low, according to a study including 371 unrelated healthy apheresis blood donors assessed by high-resolution HLA typing. In detail, whereas the allele frequency of the HLA-DQB1*02 allele was 0.3%, the frequency of DQB1*03:02 allele was actually 10.8% [13].

In addition to such a low prevalence of HLA-DQ genotypes leading to susceptibility for CD, the dietary intake of wheat in Japan is still one-third of what is observed in Western countries, although the consumption of these products has been increasing in last few years [14]. However, a very recent study by Fukunaga et al. confirmed that CD is still of low prevalence in the Japanese population, as this diagnosis was confirmed in only 0.05% of 2008 asymptomatic people having their annual check-up [15]. Unfortunately, no study is currently available about CD prevalence in Japanese children.

As for China, CD is not so rare as previously thought. Recently, Kou et al. found a prevalence of 2.85% in 246 adult patients referred for irritable bowel syndrome [16]. In this same clinical setting, another study from South China observed a 1.01% prevalence; interestingly, CD was diagnosed in 0.28% of the controls [17]. However, the meta-analysis by Juan et al. is the best source of genetic data about CD predisposition in China so far. According to this paper, published in 2013, the pooled frequencies of the DQA1*05-DQB1*02 (MHC-DQ2 genotype) and DQA1*03-DQB1*03:02 (MHC-DQ8 genotype) alleles were 3.40% and 2.10%, respectively. However, the frequency of the DQB1* 02:01 allele was as much as 10.5% overall; importantly, among all Chinese regions where the pooled data came from, significant variations were observed (from 22.04% in Xinjiang Uygur region to 2.8% in Yunnan province). Basically, this allele was more common in northern China than in the southern Chinese populations [18]. More recently, the same group reported positivity for CD serum markers in 2.19% of almost 20,000 Chinese adolescents and young students (16–25 years), who underwent routine physical examinations in two universities. However, most of them tested positive for anti-DGP IgG, whereas only 0.36% were positive for anti-tTG IgA; moreover, no information was provided about the histopathological findings of duodenal biopsy, if performed [19]. There are some other studies from China reporting anti-tTG IgA serology in Chinese patients affected with diabetes mellitus type I (DMT1) and autoimmune thyroid diseases, who were positive in 22% of cases, globally [20]. However, other than that, the only available study on pediatric CD in China described 14 patients diagnosed with CD after screening 118 children affected with chronic diarrhea. Here, CD was found in 11.9% of the study cohort and all children (1–12 years) underwent duodenal mucosal biopsy, after the detection of anti-tTG IgA (*n* = 14) and EMA (*n* = 9) positivity [21].

Unfortunately, no informative articles were retrieved from Korean peninsula and Mongolia about pediatric CD.

## 3. India

In 2002, Kaur et al. provided the first study assessing the HLA background in pediatric CD patients from India. They investigated 117 children with gastrointestinal (e.g., chronic diarrhea, abdominal pain) and extra-gastrointestinal (short stature, iron-deficient anemia, failure to thrive, etc.) manifestations who received a diagnosis of CD by intestinal biopsy: importantly, almost 100% association with DQB1*02:01 in these Indian pediatric CD patients was reported [22]. In general, according to the study by Ramakrishna et al., the prevalence of genes determining MHC-DQ2 and/or -DQ8 expression in the Indian population was around 35%; moreover, the disease prevalence was estimated to be around 1%. CD diagnosis was more frequent in the northern part of India, where the mean daily wheat intake is the highest [11].

Interestingly, an American study of nearly 500,000 duodenal biopsy samples (from all over the USA) showed that the ethnic group from the Punjab area (northern India) had the greatest prevalence of villous atrophy among all the ethnicities living in the country (3.08% vs. 1.80% for other Americans) [23]. Accordingly, in a cross sectional study involving urban and rural populations (*n* = 10,488) in the northern part of India, the overall serological prevalence of CD was 1.44%, and the overall prevalence of CD was 1.04% [24]. Moreover, Agrawal et al. provided detailed HLA immunogenetic data from 1336 unrelated healthy people from northern and northeastern India. As regards MHC-DQ2 and MHC-DQ8 alleles, these authors provided the following frequencies (highest–lowest), variable according to the region: DQA1*05:01 (11.20–16.70%), DQB1*02:01 (17.40–26.50%), DQA1*03:01 (9.50–24.10%) and DQB1*03:02 (0–5.48%) [25].

As regards the pediatric population in India, despite these findings, Sood et al. reported 0.3–0.4% CD prevalence in Punjab children and adolescents (3–17 years). However, this prevalence may have been under-estimated, as only selected groups of children were screened, based upon a structured questionnaire; thus, only these patients received a full medical assessment, including CD serology. At that time, this publication was the first large-scale study on pediatric CD (including 4347 children undergone serologic screening): it demonstrated that pediatric CD in India was more common than previously appreciated, especially in wheat-eating parts of India [26]. Conversely, Batthacharya et al. reported around 1% CD prevalence in 400 consecutive children (1–12 years) undergoing venipuncture for any reason, who were referred to the general pediatric department of a tertiary care hospital of Northern India [27]. Moreover, Srivastava et al. showed that the prevalence of CD diagnosed in the first-degree relatives (FDRs) of CD children from Northern India was 4.4%, 14 times higher than in the general population [28]. In particular, as concerns the pediatric FDRs of children diagnosed with CD, Singla et al. reported 9-fold higher CD prevalence than in the general pediatric population, with peaks in symptomatic FDRs affected with anemia and short stature [29]. Previously, Gautam et al. had investigated 63 siblings (2–15 years) of 48 CD children, and 22% were shown to have CD as well. Importantly, two third of them did not report any gastrointestinal symptoms suggestive of CD [30].

On the contrary, in Southern India CD is less frequent, which has been related to the effect of both genetic and environmental factors. Indeed, Yachha et al. highlighted that the frequency of celiac HLA-DQ predisposing alleles was much higher in Northern India (31.9%) than in Southern India (9–12.8%), where this genetic difference is coupled with a different staple diet based upon rice dishes [31].

## 4. South-East Asia

In most countries of South-East Asia, the frequency of HLA-DQB1*02 is estimated to be between 5–20% [14].

In Vietnam, CD is considered a very rare disease, although the frequency of MHC-DQ2/DQ8 alleles is not particularly low. The scarce exposure to gluten is supposed to be responsible for this epidemiological situation. Zanella et al. investigated the celiac autoimmunity in 1961 Vietnamese children having blood drawn for any reason. Around 1% of these children (median age: 5.3 years) were positive for anti-tTG IgA; however, among them, only 33% resulted to be carriers of any MHC-DQ2/DQ8 alleles, and no duodenal histopathological data were provided in this study [32].

In Thailand, Thammarakcharoen et al. assessed the frequency of HLA-DQB1*02 and DQB1*03:02 alleles, in addition to anti-tTG serology, in children with DMT1. HLA-DQB1*02 and HLA-DQB1*03:02 allele frequencies were 27% and 14% in DMT1 patients and normal controls, respectively. Only one patient was positive for anti-tTG IgA, and he was asymptomatic [33]. Therefore, the prevalence of CD screening positivity seems to be currently negligible in Thai children.

No pediatric studies were retrieved from Malaysia. However, Yap et al. reported unexpectedly high seroprevalence of CD autoantibodies (1.25%) in healthy young adults from the Malaysian population. Importantly, Malaysia has a multiracial population consisting of three major ethnic groups (Malay, Chinese, and Indian), which may affect the genetic predisposition to CD. Unfortunately, HLA haplotyping was not performed in this study and intestinal histological data were not provided [34].

Finally, no relevant studies about pediatric CD were retrieved from other countries of South-East Asia (Laos, Myanmar, Bangladesh, Singapore, Indonesia, Philippines).

## 5. Central Asia

Clinical data about the prevalence of CD in the countries of Central Asia (Kazakhstan, Kyrgyzstan, Tajikistan, Turkmenistan, and Uzbekistan) from English-language medical literature are extremely scarce.

However, a paper by Savvateeva et al. first provided an overview on CD in Russia and, interestingly, also cited some references of studies coming from the surrounding republics of Central Asia [35]. Therefore, some more information about CD in Central Asia could be obtained from Russian-language medical sources. Importantly, as for Russia specifically, most studies considered in the aforementioned article included children and, according to these authors, the estimation of pediatric CD prevalence in this country has increased from about 0.02% to 0.3% during the first decade of 2000s. Moreover, in children with consistent clinical manifestations and/or specific risk factors (e.g., autoimmune diseases, Down syndrome, etc.), CD prevalence proved to be comparable to Europe (0.94% to 15.98%, according to the specific clinical setting). Additionally, Savvateeva et al. reported data on the polymorphic variants of HLA-DQA1 and -DQB1 genes in CD patients from the cities of Tomsk and Krasnodar (which are located in Russia, north and west of Kazakhstan respectively) and from Yakutia (Eastern Russia). These studies (all published in Russian) suggested that the allelic frequencies of CD-related HLA-DQ genes in those populations may be comparable to those observed in Europe [35].

We traced back some of the primary sources describing those data, which were published in Russian-written journals. The study by Kurtanov et al. examined 37 children (1–18 years) affected with gastrointestinal symptoms; here, an interesting finding was a relative abundance of CD patients carrying the MHC-DQ8 genotype, which was found in > 30% of Yakuts patients [36]. Tlif et al. authored a study from Krasnodar which included 110 children with DMT1 and 654 controls, whereby the respective frequencies of CD predisposing HLA-DQ alleles were as follows: DQA1*05:01 (28.6% and 29%), DQB1*02:01 (33.6% and 19.5%), DQA1*03:01 (38.2% and 12.2%), and DQB1*03:02 (31.6% and 8.3%). Among these DMT1 patients, 32 children were diagnosed with CD and they showed the following genotype distribution: DQA1*05:01 (40.6%), DQB1*0201 (35.9%), DQA1* 03:01 (9.4%), and DQB1*03:02 (7.8%). Therefore, this study showed a relative proportion between MHC-DQ2 and MHC-DQ8 alleles that seems to follow the same trend described in Europe [37].

The only study from Kazakhstan (again, cited by Savvateeva et al.) was authored in Russian language by Sharipova: this author described patients from the Almaty region, which is located in the southern part of the country. Starting from 17,800 pediatric patients registered in two clinics of the city, this author considered 6,380 children who displayed gastrointestinal symptoms and/or malnutrition, or had autoimmune diseases (such as thyroiditis and DMT1) or a sibling affected with CD. Among them, the author selected a group of 1,220 children as ‘at risk for CD’ based on the clinical examination; however, no precise inclusion criteria were described. One out of three children were then randomly chosen to undergo assessment of antigliadin antibodies (AGA) IgG and IgA titers, meaning that around 400 children received this screening. Finally, 86 children were positive to AGA IgA and/or IgG, and 74 of them (aged 3–15 years) were reported as affected with CD according to their histopathological results (however, once again, no detailed description was provided). Based on this study, the CD detection rate was 1 case in every 18 screened children, which allowed this author to estimate a CD prevalence of 1:262 children in Kazakhstan [38]. It is evident that such an experimental approach had several limitations, as selection and analysis bias may have been introduced in this numerical estimation, in addition to the poorly sensitive and specific screening protocol. Indeed, very recently Verma et al. confirmed that almost 40% of anti-tTG IgA positive celiac children are missed when using a screening approach based on AGA IgA only [39].

Moreover, this study from Kazakhstan also reported the genetic analysis of MHC-DQ2 and MHC-DQ8 related alleles: these results were not displayed in detail, but it is very interesting to notice that the frequency of these susceptibility alleles in CD children was apparently approximately 60% only [38]. Actually, this finding might raise some further concerns about the diagnosis of CD, considering the very high, almost absolute, negative predictive value associated with the complete absence of any of the alleles coding α and β chains of MHC-DQ2 and -DQ8 heterodimers [3]. However, even CD children included in the available studies from Central and Eastern Russia were shown to carry these alleles with a significantly lower frequency than expected, namely around 80% [35].

As regards other countries of Central Asia, even the research in the Russian literature was scant. However, Abduzhabarova et al. published an article in English providing some data about the HLA immunogenetic profile in 54 Uzbek children (aged 1–14 years) diagnosed with CD. Although it was not possible to retrieve the raw data, 36 CD children (around 70%) were shown to carry MHC-DQ2 alleles, but data related MHC-DQ8 alleles were not available. However, an interesting point is that around 36% of their 109 healthy control children were carriers of MHC-DQ2 alleles, which is consistent with prevalence data observed in the European general population [40].

## 6. Conclusions

Except for Indian children, epidemiological studies and clinical research about pediatric CD from Central and East Asia are lacking or poor. However, there is initial evidence that the MHC-DQ2 and MHC-DQ8 immunogenetic background is not so unusual in several populations from these areas as previously or commonly thought. Indeed, in South-East Asia the frequency of HLA-DQB1*02 is estimated to be comprised between 5–20%, and in Central Asia the prevalence of HLA-DQ alleles predisposing to CD may be even higher. As concerns the disease occurrence, the exposure to the environmental trigger, namely gluten, currently makes the difference among different countries in Asia. Whereas in Vietnam and Thailand the CD prevalence is still negligible because of the rice-based dietary regimen, in Russia and in the Republics of Central Asia the occurrence of CD seems to be growing, given the large consumption of wheat. Indeed, the wheat consumption per person per year is estimated to be lower than 25–50 kg in most regions of South-East Asia, whereas it does exceed 150 kg in Kazakhstan, Uzbekistan, Tajikistan, Kyrgyzstan, and Turkmenistan [12].

However, such a CD epidemiological burden has not been clearly evidenced yet, probably because the disease is actively sought only in the presence of (severe) gastrointestinal manifestations, and the access to CD diagnostic procedures is still limited for several reasons. Therefore, it is essential to implement cost-effective diagnostic protocols that can allow the rest of the ‘celiac iceberg’ to emerge, considering the potential and persistent health consequences related to the under-diagnosis of CD in children. Indeed, this under-diagnosis may also represent an important issue of public health, especially in those countries where the birth rate is significantly higher and the population is relatively younger than in Western countries.

In conclusion, it is likely that CD is not so uncommon, even in several Asian countries other than India and Western Asia. Therefore, there is urgent need for population-based studies on the prevalence of CD in these countries, especially in Russia and Central Asia, in order to increase the awareness of this disease and to improve the diagnostic strategy.
